# Intraoperative Dexmedetomidine Promotes Postoperative Analgesia in Patients After Abdominal Colectomy: A Consort-Prospective, Randomized, Controlled Clinical Trial

**DOI:** 10.1097/MD.0000000000001514

**Published:** 2015-09-18

**Authors:** Dong-Jian Ge, Bin Qi, Gang Tang, Jin-Yu Li

**Affiliations:** From the Department of Anesthesiology, Huai’an First People's Hospital, Nanjing Medical University, 6 Beijing Road West, Huai’an, Jiangsu, 223300, P. R. China.

## Abstract

Surgery-induced acute postoperative pain may lead to prolonged convalescence. The present study was designed to investigate the effects of intraoperative dexmedetomidine on postoperative analgesia following abdominal colectomy surgeries. Eighty patients scheduled for abdominal colectomy surgery under general anesthesia were divided into 2 groups, which were maintained using propofol/remifentanil/dexmedetomidine (PRD) or propofol/remifentanil/saline (PRS). During surgery, patients in the PRD group had a lower bispectral index (BIS) value, which indicated a deeper anesthetic state, and a higher sedation score right after extubation than patients in the PRS group. During the first 24 hours post surgery, PRD patients consumed less morphine in patient-controlled analgesia (PCA) and had a lower score in the visual analog scale (VAS) testing than their controls from the PRS group. Intraoperative administration of dexmedetomidine appears to promote the analgesic property of morphine-based PCA in patients after abdominal colectomy.

## INTRODUCTION

Postoperative pain is one of the key causes of prolonged convalescence following abdominal surgery.^1–5^ Opioid-based PCA is well-established and widely used in postoperative analgesia due to their potent analgesic effects.^6^ Currently, the main challenge in PCA is to reduce opioid consumption, and related side effects such as nausea, vomiting, itch, etc.

Anesthesia management may modulate surgery-induced pain.^7^ Recent clinical studies reported that the highly selective alpha-2 adrenergic receptor (α2-AR) agonist dexmedetomidine promotes the analgesic effect and prolongs the analgesic time of local anesthetics even to 24 hours after dental and osteopathic surgeries.^3,7^ These studies investigated the synergic action of intraoperative dexmedetomidine with local anesthetics on surgery-induced acute pain during or following surgeries.^3,7^ The evidence above suggested that intraoperative application of DEX may be helpful to promote the analgesic effect of PCA postoperatively, which will benefit patients with surgery-induced pain. DEX had been reported to reduce morphine consumption and related side effects in different surgeries both in children and adults.^8–10^ These evidence leads us to a hypothesis that dexmedetomidine might have a long-lasting pro-analgesic effect.

However, some side effects, such as hypotension and bradycardia, have limited the clinical applications of DEX under conditions without professional monitorings. Therefore, in the present study, we hypothesized that intraoperative DEX may improve the analgesic effect of morphine-based PCA and reduce morphine consumption and related side effects in patients following abdominal colectomy.

## METHODS

### Subjects

**FIGURE 1:**
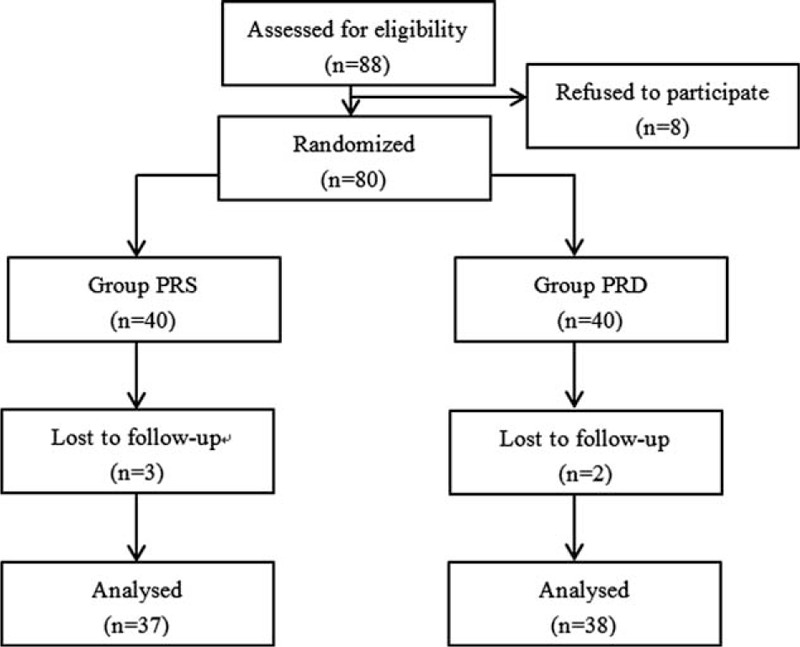
Flow diagram of the study.

**FIGURE 2:**
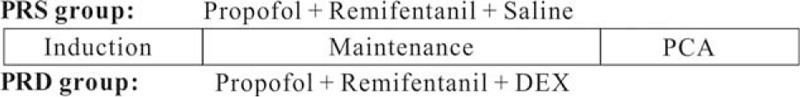
Schematic of anesthesia and post-operation analgesia. Patients received same treatments for induction and PCA (see *Methods* part). Patients in both groups received anesthesia maintenance with propofol, remifentanil, and saline (PRS group) or dexmedetomidine (PRD group). PCA = patient-controlled analgesia, PRD = propofol/remifentanil/dexmedetomidine, PRS = propofol/remifentanil/saline.

This study was approved by the Institutional Medical Ethics Committee of Nanjing Medical University and was in accordance with the approved guidelines. Informed consent was obtained from all subjects. The sample size of the study was calculated according to previous studies,^11,12^ and based on a pilot study. Twenty-one patients in each group were required to detect a difference of “1 over 10” in the VAS score with a power of 0.8 and type I error of 0.05.^11^ Eighty of 88 qualified patients (8 patients refused) were enrolled, and randomly assigned into PRS (n = 37, 3 patients were lost because of noncooperation), and PRD (n = 38, 3 patients were lost because of noncooperation) group using the computer-generated randomized table (Figure 1). The PRS and PRD patients received propofol, remifentanil, and saline or dexmedetomidine for general anesthesia maintenance, respectively (Figure 2). The maintenance syringe pumps were prepared by a different anesthesiologist to make this study a randomized, double-blinded investigation. Patients matching the following criteria were included in this study: between 40 and 75 years old; American Society of Anesthesiologists (ASA) grade I or II; weight 55 to 85 kg; height 150 to 190 cm. Patients were excluded if they had ischemic heart diseases; opioid addiction, long-term alcohol abuse, long-term smoking history, sedative–hypnotic drug(s); obesity (BMI > 30); postoperative nausea and vomiting history; or neuropsychiatric diseases and related treatment history. Patients were instructed to the use of the visual analog scale (VAS; 0, no pain, and 10, worst pain possible) and the *i.v.* PCA pump (50 mg morphine and 8 mg ondansetron in 100 ml saline, every pump press leads to a 2 ml of infusion).

## ANESTHESIA

On arrival, electrocardiography, blood pressure, oxygen saturation, and the bispectral index (BIS) were monitored every 5 minutes. A BIS value of <60 was used to adjust the titration of anesthetics on the basis of amnesia. For induction, patients from the both groups received midazolam (0.05 mg/kg), remifentanil (2–5 μg/kg), propofol (4–6 mg/kg), and cisatracurium (0.2 mg/kg). Immediately after intubation, patients were ventilated with an oxygen and air mixture (FiO2 = 0.4) with a PetCO_2_ at 30 to 35 mm Hg, intravenous infusion was switched to maintenance syringe pump at a rate of 50 to 80 μg/kg/min for propofol, 0.15to 0.2 μg/kg/min for remifentanil, and 0.4 μg/kg/h for dexmedetomidine. Cisatracurium (0.05 mg/kg) was intermittently used for muscle relaxation. Patients were awoken and extubated followed by sedation evaluation using the Ramsay sedation scale.

### Data Collection

Patient demographic information was collected on admission. Hemodynamic indexes and BIS were recorded during surgery every 5 min, and data from selected timepoints used for analysis. Postoperative pain at rest and on movement was evaluated with VAS. Subjects that received rescue morphine in the PACU had the rescue morphine included in the total consumption of postoperative PCA morphine. PCA pump pressing number and adverse effects after surgery were noted.

### Statistics

All data in the present study were analyzed with GraphPad Prism 5.0 software. Parameters such as age, weight, operation time, anesthesia time and PACU stay time, pump-press number, and morphine consumption were compared between the 2 groups by unpaired Student's *t* test. heart rate (HR), MBP, VAS, and BIS at different time points were compared between the 2 groups with 2-way ANOVA followed by Bonferroni post-test. ASA grade and postoperative adverse effects were analyzed with Fisher's test. All data with *P* < .05 were considered significant.

## RESULTS

### Demographic Data and Surgery/Anesthesia-Related Information

Eighty qualified patients were enrolled, and randomly assigned into PRS (n = 37, 3 patients were lost because of noncooperation) and PRD (n = 38, 2 patients were lost because of noncooperation) groups using a computer-generated randomized table. Patients from the both groups have comparable demographic and surgery/anesthesia-related variables, including age, weight, BMI, ASA class, operation time, anesthesia time, and PACU stay time (Table 1).

**TABLE 1:**
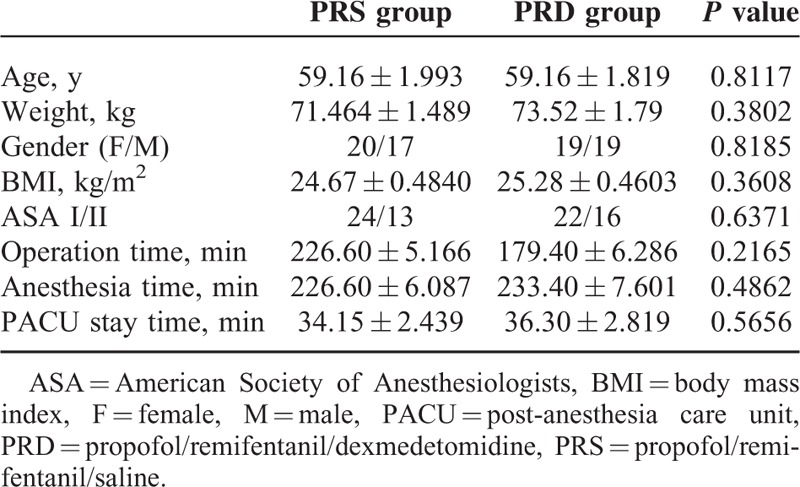
Basic Demographic Data and Surgery/Anesthesia-Related Information. Data Shown as Mean ± SEM

**FIGURE 3:**
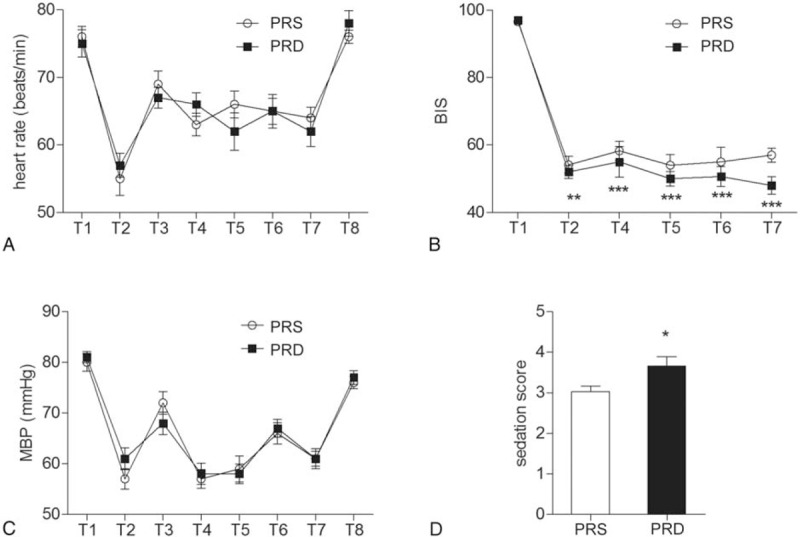
Heart rates, MBP, BIS value, and Romsay sedation score. (A) Heart rates at different time points. (B) MBP at different time points. (C) BIS values at different time points, ^∗∗^*P* < 0.01, ^∗∗∗^*P* < 0.0001. (D) Romasy sedation scale score right after extubation, ^∗^*P* < 0.05. For Figure 2A–C: T1: baseline, T2: induction, T3: intubation, T4 to T7: 10, 30, 60, and 90 minutes after intubation, T8: 24 hours after surgery. BIS = bispectral index, MBP = mean blood pressure.

The 2 groups were also comparable with respect to their baseline mean blood pressure (MBP) and HR, followed by a decrease-induced by induction and sharp increase-evoked by intubation. Subsequently, the MBP and HR were maintained at a lower level than baseline to extubation. And 24 hours after surgery, the MBP and HR returned to the baseline level (Figure 3A and B).

### Anesthesia depth evaluation

Anesthesia depth was monitored with BIS. Significantly, patients form the PRD group had a lower BIS value when compared to the PRS group (Figure 3C, ^∗∗^*P* < 0.01, ^∗∗∗^*P* < 0.0001), which indicated a deeper anesthesia state. The PRD group also had a higher immediate Ramsay sedation score after extubation when compared to their controls in the PRS group (Figure 3D, ^∗^*P* < 0.05).

### Postoperative PCA Evaluation

**FIGURE 4:**
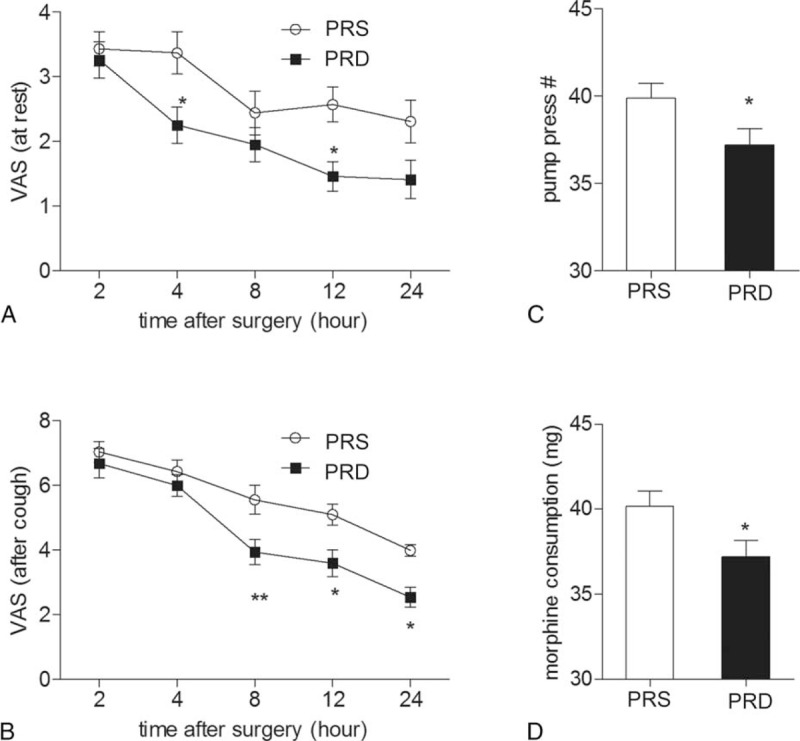
24 hours PCA evaluation and morphine consumption. (A) VAS pain score at rest at different time points in the 2 groups, ^∗^*P* < 0.05. (B) VAS pain score after coughing at different time points in the 2 groups, ^∗^*P* < 0.05, ^∗∗^*P* < 0.01. (C) and (D) show pump press numbers and morphine consumption during the first 24 hours following surgery, ^∗^*P* < 0.05. PCA = patient-controlled analgesia, VAS = visual analog scale.

After surgery, the patients received a morphine-based PCA pump. Postoperation pain was assessed with VAS, and the pain-induced pump press number, and morphine consumption were noted. During the first 24 hours, patients from the PRD group had a lower VAS score both at resting (Figure 4A, at time point of 4 and 12 hours following surgery, ^∗^*P* < 0.05) and movement states (after coughing, Figure 4B, at time point of 8, 12, and 24 hours following surgery, ^∗^*P* < 0.05, ^∗∗^*P* < 0.01) compared to the PRS group. Patients from the PRS group also had a higher pump press number and more morphine consumption than the PRD group (Figure 4C and D, ^∗^*P* < 0.05).

### Postoperative adverse effects

No difference was observed in the postoperative adverse effects between the 2 groups during the first 24 hours. PRD patients had a trend of suffering from less adverse effects such as nausea, vomiting than those from the PRS group (Table 2).

**TABLE 2:**
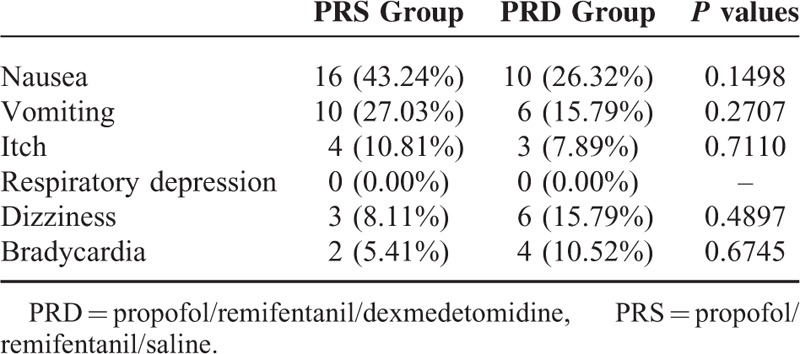
Postoperative Side Effects from Patients in the 2 groups. Data shows the positive number and percentage of patients

## DISCUSSION

It is widely known that patients undergoing abdominal colectomy experience severe acute postoperative pain,^4,13–15^ which may lead to chronic pain. Opioids, especially morphine-based patient controlled analgesia, are widely used for pain control following abdominal surgeries.^16,17^ To combat the side effects, such as nausea, vomiting, itch, and so on, there has been a pursuit for novel drugs, or more information regarding combining currently available drugs to reduce the morphine consumption. Alpha 2 receptor agonists, such as clonidine (α2R:α1R ratio is 200:1), has been used as pain treatment for decades.^18,19^ Recent study reported that α1 receptor activation encountered α2R-related analgesia, which suggested that an agonist with a higher α2 R selectivity would show a more potent analgesic effect and would be more suitable for pain treatment.^20^ DEX is an α2R agonist developed in the 1990s and was first used for a short-term sedative in the intensive care unit.^7,21^ Clinical studies have confirmed its potential as an adjuvant of local analgesics for pain treatment, mostly during the acute perioperative settings. This suggests that DEX might act as a novel candidate in surgery-induced acute pain control.^16^ In the present study, we combined dexmedetomidine with propofol and remifentanil to maintain the general anesthesia in patients undergoing abdominal colectomy surgery. Patients from the PRD group that received intraoperative dexmedetomidine had a lower score in both resting VAS test and after coughing VAS test, and also these patients had smaller pump-press numbers, and consumed less morphine than those from the PRS group. These data suggested that intraoperative DEX are useful to promote morphine-based PCA and had a clear mophine-sparing effect in patients after abdominal colectomy. The analgesic and opioid-sparing effect of dexmedetomidine have been well described in previous studies both in adult and children.^8–10^ Similar to the present data, these studies reported significantly lower VAS, morphine consumption, and morphine demands. Together with these findings, the present study indicated that intraoperative administration of dexmedetomidine is a potential way to be used to promote morphine-based PCA following abdominal surgery.

As expected, we also observed significant lower BIS values in the PRD group during anesthesia, and higher sedation score right after extubation, which was consistent with previous reports,^17^ and indicates that intraoperative dexmedetomidine provided a more stable anesthesia without changing haemodynamic characters.^15^ And this sedation state might be a positive factor contributed to the analgesia-promoting and morphine-sparing effect of intraoperative administration of DEX.

So far, the mechanisms underlying the long-lasting analgesic or pro-analgesic effects of dexmedetomidine are still unknown. Dexmedetomidine was first introduced into clinical use as a short sedative since it is a fast-metabolized chemical with a short plasmatic half-time of 2 to 2.5 hours.^16^ There are several possibilities responsible for the long-lasting analgesic effect: unlike the sedation effect, dexmedetomidine is using a different α2AR-dependent downstream mechanism to act as an analgesic; another reason might be that dexmedetomidine prolongs the analgesic time and the analgesic effect of other analgesics. Although an animal study reported that its analgesic property could be neutralized by α2AR antagonist,^22^ we cannot completely exclude the remote possibility that dexmedetomidine is also using α2AR-independent mechanisms to show its analgesic effects.

And in the present study, we found that dexmedetomidine induced sedation and analgesia without increasing the risks of opioid-related side effects, such as respiratory depression, which is consistent with previous reports. We also saw a decreasing trend of postoperative nausea and vomiting. At this point, a relatively smaller sample size might be a limitation of the present study; future large sample studies should be done to verify its effects on morphine-and surgery-related side effects, such as nausea and vomiting.

Taken together, maintenance with dexmedetomidine (0.4 μg/kg/h) provided a more stable anesthesia without changing hemodynamic characters and is useful and suitable to promote the morphine-based PCA following abdominal colectomy. Generally, patients with acute postoperative pain might benefit from intraoperative application of dexmedetomidine.
